# Influenza forecast optimization when using different surveillance data types and geographic scale

**DOI:** 10.1111/irv.12594

**Published:** 2018-08-21

**Authors:** Haruka Morita, Sarah Kramer, Alexandra Heaney, Harold Gil, Jeffrey Shaman

**Affiliations:** ^1^ Department of Environmental Health Sciences Mailman School of Public Health Columbia University New York City New York; ^2^ Marion County Public Health Department Indianapolis Indiana

**Keywords:** forecasting, influenza, optimization, surveillance data

## Abstract

**Background:**

Advance warning of influenza incidence levels from skillful forecasts could help public health officials and healthcare providers implement more timely preparedness and intervention measures to combat outbreaks. Compared to influenza predictions generated at regional and national levels, those generated at finer scales could offer greater value in determining locally appropriate measures; however, to date, the various influenza surveillance data that are collected by state and county departments of health have not been well utilized in influenza prediction.

**Objectives:**

To assess whether an influenza forecast model system can be optimized to generate accurate forecasts using novel surveillance data streams.

**Methods:**

Here, we generate retrospective influenza forecasts with a dynamic, compartmental model‐inference system using surveillance data for influenza‐like illness (ILI), laboratory‐confirmed cases, and pneumonia and influenza mortality at state and county levels. We evaluate how specification of 3 system inputs—scaling, observational error variance (OEV), and filter divergence (lambda)—affects forecast accuracy.

**Results:**

In retrospective forecasts, and across data types, there were no clear optimal combinations for the 3 system inputs; however, scaling was most critical to forecast accuracy, whereas OEV and lambda were not.

**Conclusions:**

Forecasts using new data streams should be tested to determine an appropriate scaling value using historical data and analyzed for forecast accuracy.

## 1 INTRODUCTION

Seasonal influenza outbreaks in the United States cause 140 000‐710 000 hospitalizations and 12 000‐56 000 deaths annually.^1^ These epidemics regularly recur during winter, but outbreak characteristics, such as peak timing and intensity, vary considerably. These year‐to‐year differences make timely healthcare preparedness measures, including resource allocations, hospital staffing, and dissemination of alerts, challenging. As a result, influenza outbreaks can cause serious strain on healthcare infrastructure and personnel, which lowers quality of patient care. A study reported increases in physician and emergency department (ED) visits for respiratory illnesses during influenza outbreak peak weeks of 7% and 9%, respectively.^2^ An increase in emergency medical services diversion, an indicator of ED overcrowding, was also reported during peak influenza periods.^3^ Furthermore, Schilling et al found increased in‐hospital mortality due to ED overcrowding during high‐influenza periods.^4^

Real‐time influenza forecasts can help planning for patient surges and could reduce associated costs, patient morbidity, and patient mortality. These forecasts provide 12‐week or more lead predictions of future influenza incidence including predictions of peak timing and magnitude estimates for the ongoing outbreak. Earlier knowledge of outbreak dynamics can give healthcare providers more time to enact preparedness measures to combat ED overcrowding, as well as resource and staff shortages.^5^, ^6^ Whereas these predictions have great potential to lessen influenza mortality and morbidity, high‐quality and accurate influenza surveillance data are needed to generate skillful forecasts.

Many existing influenza forecasting systems rely upon data from the US Centers for Disease Control and Prevention (CDC). CDC provides national‐, regional‐, and, just recently, state‐level syndromic surveillance data for influenza‐like illness (ILI), as well as laboratory‐confirmed cases of influenza, including specific strains.^7^ ILI is a nonspecific metric that includes persons who present to an outpatient clinic with a fever of 37.8°C or greater and a cough and/or sore throat.^8^ Although CDC provides these datasets publicly, their geographic scale is limited to the United States overall, large US regions, and states. Forecasts generated at a finer geographic scale could potentially provide more relevant localized information for influenza outbreak preparedness.

Google Flu Trends (GFT) was an alternate source of influenza surveillance data that produced estimates of ILI at state and city spatial resolutions. Until July 2015, Google produced GFT, which used an algorithm to estimate daily ILI based on Google online search activity and autoregressive terms.^9^ GFT was produced in real time, unlike CDC ILI, which is published weekly with a 6‐ to 12‐day delay. During its period of real‐time publication, the GFT data stream was used to generate accurate predictions of future influenza incidence and outbreak dynamics.^10^, ^11^, ^12^, ^13^ Nsoesie et al used GFT to forecast influenza peak timing in Seattle, Washington, 5‐6 weeks before the peak.^14^ Additionally, Shaman and Karspeck developed a real‐time seasonal influenza forecast for New York City using GFT and generated skillful predictions of peak timing 7 weeks before the true peak.^15^

However, GFT had well‐documented limitations: The algorithm did not capture the first wave of the 2009 A/H1N1 influenza pandemic^9^, ^16^ and inflated the magnitude of the A/H3N2 epidemic during the 2012‐13 season.^9^ Recently, several studies have presented new algorithms using online search queries,^12^, ^17^ tweets,^18^, ^19^ Wikipedia access logs,^20^, ^21^ or other public generated content;^22^, ^23^, ^24^ however, as these algorithms are all trained to estimate CDC ILI, which until fall 2017 was only available at national and regional geographic scales, forecast of more local influenza activity is often difficult.

Influenza surveillance data are collected by local health departments across the United States in many different forms, such as cases of ILI, ED visits for influenza, and influenza‐related deaths. Some of these data are provided to CDC as part of national influenza surveillance, and many local health departments post weekly influenza reports online to provide information on the current influenza burden to health professionals and to the general public. Despite widespread existence of these local data and their potential for producing local influenza forecasts, relatively few studies have used surveillance data from state, county, or city departments of health for modeling influenza outbreaks.^25^ A recent study generated accurate forecasts of influenza peak timing in Melbourne, Australia, using a range of local syndromic and laboratory‐confirmed data streams.^26^ However, we are unaware of analogous studies in the United States.

Given that hospital and public health decision‐making in response to influenza is often made at local geographic scales, it is important that accurate, operational, real‐time forecasting systems be developed and validated at those scales. Different surveillance data (eg, hospitalizations, virology, ILI, and mortality) provide different estimates of influenza activity and outbreak dynamics. For example, increases in ILI may signal the start of an epidemic, whereas upticks in hospitalizations and mortality may be an indicator of activity *and* virulence of circulating strains. Predictions, in turn, may represent dynamics specific to a particular surveillance data type, which delineates how those predictions might be used to inform interventions. More work is thus needed to investigate differences in forecast accuracy and utility when incorporating different local surveillance data types.

Here, we investigate these issues by testing an influenza forecast model using 6 types of surveillance data from state and county departments of health in Arizona and Indiana. Overall, this work aims to determine the feasibility of using local influenza surveillance data to generate accurate real‐time forecasts. Specifically, we sought to answer the following research questions: (a) Does our real‐time influenza forecasting model perform equally well using different types of local surveillance data? (b) Does model specification need to be adjusted for different types of local surveillance data? and (c) Which model specifications perform best for different types of local surveillance data? Understanding the differential utility of local surveillance data types will allow researchers to intelligently use these data streams in forecasting efforts, improving public health and medical response to influenza outbreaks at local scales.

## 2 METHODS

This study employed a humidity‐driven model‐inference influenza forecast system. There are 3 main components of this system: (a) real‐time observations of influenza incidence; (b) a dynamic state‐space model describing the propagation of influenza through a population; and (c) a data assimilation method. The system uses real‐time observations and a data assimilation method to iteratively update dynamic model state variables and parameters to better match ongoing outbreak dynamics. This process optimizes model state space, enables inference of critical epidemiological parameters, and facilitates generation of accurate real‐time ensemble forecasts. The form and function of each system component are further described below.

### 2.1 Influenza surveillance data

Six distinct influenza surveillance time series from two states, Indiana and Arizona, were used (Table 1). Indiana data were obtained from the Marion County Public Health Department and the Indiana State Department of Health. Arizona data were obtained from the Maricopa County Department of Public Health. Data streams are described further below, and methods used to process ILI data specifically, and to process all data are available in Supporting information.

**Table 1:** Details of surveillance data and parameter values used for forecasts

Geographic scale		Seasons (excluding 2008‐10)	Surveillance type	Scaling values used	OEV	Lambda
Arizona	County	2004‐2014	Sentinel ILI	0.8, 1, 2, 3, 4, 5, 10	0, 1, 2	1.00, 1.01, 1.02, 1.03
			Emergency Dept. ILI	0.1, 0.2, 0.3, 0.4, 0.5, 0.6, 0.7		
			Laboratory‐confirmed	100, 250, 500, 750, 1000, 1250, 1500		
			P&I Deaths	0.1, 0.2, 0.3, 0.4, 0.5, 0.6, 0.7		
Indiana	State	2002‐2015	ILI	0.5, 1, 1.5, 2.0		
	County	2005‐2014	ILI	15, 20, 25, 30, 35, 40		

P&I, pneumonia and influenza.

#### 2.1.1 Indiana

Influenza‐like illness data were obtained at two geographic scales in Indiana: (a) ILI data of Marion County residents seen at Marion County hospitals, and (b) outpatient ILI data from clinics enrolled in the US Outpatient Influenza‐like Illness Surveillance Network (ILINet).

#### 2.1.2 Maricopa County, Arizona

The Maricopa County Department of Public Health provided 4 different influenza surveillance data streams: (a) weekly ILI cases aggregated from sentinel site clinics across the county; (b) weekly ILI cases from EDs; (c) weekly laboratory‐confirmed cases of influenza, comprised of patients who were tested and confirmed to have influenza; and (d) records of pneumonia and influenza (P&I) deaths.

### 2.2 SIRS model

The dynamic, compartmentalized model was a susceptible‐infected‐recovered‐susceptible construct with absolute humidity forcing that describes influenza transmission in a perfectly mixed population.^15^ Model equations and details can be found in Supporting information.

### 2.3 Model‐data assimilation methods

The data assimilation method used is the ensemble adjustment Kalman filter (EAKF).^27^ The SIRS model was initiated at the beginning of the flu season, integrated forward in time, and trained with the influenza surveillance observations using the EAKF algorithm up to the week of forecast initiation. Further details on EAKF can be found in Supporting information.

### 2.4 Retrospective forecasts

The humidity‐forced SIRS‐EAKF model was used to generate forecasts for each available influenza season, excluding 2008‐2009 and 2009‐2010 pandemic years to focus our predictions on seasonal influenza. The model was initiated early in October (MMWR Week 40) before influenza activity typically starts with a random selection of model state variables (S and I) and parameters (*L*,* D*,* R*_0 max_, *R*_0 min_). For each season, the model was run 10 times using a 300‐member ensemble. Each week, influenza data were assimilated using the EAKF algorithm and a new posterior ensemble was generated, which was then propagated to the next weekly observation at which point the assimilation process was repeated. This iterative optimization was repeated up to the point of forecast initiation (MMWR Week 45), after which the posterior ensemble was propagated into the future to the end of the season without further training, thus generating a forecast. For each subsequent week during the season (ie, through Week 65), the entire process was repeated with assimilation until each week of forecast initiation (eg, 46, 47) followed by ensemble forecast generation. A plot depicting components of a forecast in a given week can be found in (Figure S1).

Each ensemble member was initiated with a random set of state variables and parameters selected using a Latin hypercube sampling strategy from a predetermined range of each variable and parameter, similar to Shaman and Karspeck.^15^ Parameter ranges were 2 ≤ *L *≤* *10, 2 ≤ *D *≤* *7, 1.3 ≤ *R*_0 max _≤ 4, and 0.8 ≤ *L *≤* *1.3.

### 2.5 Varying relevant system inputs

An objective of this study was to determine how optimal system input values vary in accordance with different surveillance data types. We focused on 3 system inputs: observational error variance (OEV), scaling factor, and inflation (details below). These parameters must be specified by the forecaster, whereas other parameters, such as the basic reproductive number, infectious period, and average duration of immunity, are estimated objectively using data assimilation. Each parameter was assigned several possible values, and retrospective forecasts were generated using all possible combinations of these parameter values. This analysis was executed for each surveillance data type, and results were evaluated as explained later in this section. Table 1 shows parameter values used for each data stream.

#### 2.5.1 *Observational error variance*

Observational error variance is an input for the EAKF algorithm that represents the error associated with observations. Given the variation in the collection and measurement of different data types, we can reasonably expect error variance differences as well. Shaman et al^13^ represented OEV for ILI+ (GFT ILI multiplied by CDC influenza positive proportions) observations at week *k* as:

(1) $${\text{OEV}}_{k}=\left[1\times 10^{5}+\frac{{\left(\sum_{j=k-3}^{k-1}\frac{{\text{ILI}}_{j}}{3}\right)}^{2}}{5}\right]$$

Here, ILI_*j*_ is the influenza estimate for week *j*. OEV in this structure fluctuates in proportion to the sum of the prior 3 weeks of observations. To test different OEV structures for our data types, we maintained the same OEV structure but divided the total variance by 10^*x*^ (Equation 2). We tested *x *=* *0, 1, and 2.

(2) $${\text{OEV}}_{k}=\frac{\left[1\times 10^{5}+\frac{{\left(\sum_{j=k-3}^{k-1}\frac{{\text{ILI}}_{j}}{3}\right)}^{2}}{5}\right]}{10^{x}}$$

#### 2.5.2 *Multiplicative inflation factor*

One potential challenge associated with use of the EAKF algorithm is filter divergence, which can cause model estimates to stray from the true trajectory. This occurs when prior ensemble spread becomes spuriously small so that ensemble prior moments receive too much weight relative to observations. To counteract filter divergence, we applied a multiplicative inflation, *λ*, to the variance of the observed state variable, influenza incidence, before each weekly observation. Optimal values of *λ* may differ between data types. Hence, we varied this parameter from 1.00 to 1.03.

#### 2.5.3 *Scaling of influenza data*

Another challenge related to the EAKF system is that influenza surveillance data do not map directly to the SIRS model output. The SIRS model simulates per capita incidence; however, the ILI surveillance data capture incidence among people seeking medical care for Maricopa County and Indiana State and among the total population for Marion County, laboratory data capture positive cases among the total population of Phoenix, and deaths capture numbers of P&I deaths among those who died of all causes. To account for these differences, observations were mapped into per capita incidence using a scaling factor *γ*, per Shaman et al^13^ Specifically, by Bayes’ theorem

(3) $$p\left(i\right)=\frac{p(m)}{p(m|i)}p(i|m)\approx \gamma \left(\text{flu obs}\right)$$

*p* (*i*) is the probability of having influenza, that is, incidence as estimated by the SIRS model; *p* (*i*|*m*) is the probability of having influenza among those seeking medical attention, which is estimated by observations; *p* (*m*)is the probability of seeking medical attention; and *p* (*m*|*i*) is the probability of seeking medical attention given one has influenza. We define the scaling term, $\gamma =\frac{p(m)}{p(m|i)}$, so that *γ* represents the probability of seeking medical care divided by the probability of seeking medical care given you are infected with influenza. In this equation, *p* (*i*) remains the same regardless of data type; however, probabilities of seeking medical care and testing positive for influenza will likely differ based on type of care being sought and type of diagnosis.^13^ Hence, scaling factors will vary depending on type of surveillance data used. Given the varied data types employed, a wide range of scaling values were tested, ranging from 0.1 to 750.

### 2.6 Evaluation of retrospective forecasts

For each data type, the model system was run using combinations of OEV, inflation factor, and scaling listed in Table 1. Results were evaluated using several metrics to determine which parameter combinations generated the most accurate forecasts for each individual data stream. Methods used for forecast evaluation were peak timing accuracy, peak intensity accuracy, root mean square error (RMSE), mean absolute percentage error (MAPE), and correlation of forecasted incidence with observations. In calculating each of these metrics, forecasted values were compared to observations of the corresponding data stream. Accurate forecasted peak timing was defined as being within ±1 week of the observed peak timing.^28^ Accurate forecasted peak intensity was defined as within 25 percent (±12.5%) of the observed peak intensity. Lower values of RMSE and MAPE, and higher correlations, indicated more accurate forecasts. Information on how RMSE and MAPE were calculated can be found in Supporting information.

Initial assessment of parameters yielding the most accurate forecasts was performed by visual inspection. Specifically, boxplots were generated for RMSE, MAPE, correlations, and errors in peak week and intensity predictions. These were used to determine ranges of scaling, lambda, and OEV that appeared to be most accurate. These ranges were chosen separately for each data stream. Then, for these ranges of parameters, accuracy proportions (proportion of accurate ensemble forecasts) for peak timing and intensity were compared using a chi‐square test of equal proportions. RMSE, MAPE, and correlation were compared using ANOVA, and Tukey's honestly significant difference (HSD) test with a Bonferroni correction. This test was used to test all pairwise comparisons of means and allowed us to, within each data type, compare error measures calculated for each parameter set against one another. In most cases, this reduced the number of parameter sets considered to be best for a given data type. However, it did not necessarily yield a single optimal parameter set for each data type for two reasons: first, because several datasets that did not yield statistically significantly differences in a single accuracy metric were considered to be equally “optimal,” and second, because parameter set or sets best forecasting influenza by one metric were not necessarily the same as those sets yielding the most accurate forecasts by other metrics. Finally, a similar approach was used to evaluate differences in forecast accuracy by data type. That is, after optimal parameter sets were chosen for each individual data type, forecasts using each data type's respective optimal parameter set were compared using the peak timing and intensity accuracy metrics.

Peak timing and intensity accuracy were also compared between parameter sets averaged over the course of the epidemic, as well as using 3‐week lead time bins. Specifically, metrics were compared for forecasts predicted to be 6 and 4 weeks before the epidemic peak, between 3 and 1 week before the peak, and between the peak itself and 2 weeks after the peak.

Historical expectance and likelihood were calculated to determine whether forecasts generated with SIRS‐EAKF model‐inference system outperform those estimated from historical data (methods in Supporting information). Finally, leave‐one‐out cross‐validation was carried out to determine whether optimal parameter sets were consistent across seasons (methods and results in Supporting information).

## 3 RESULTS

### 3.1 Description of observations

**Figure 1:**
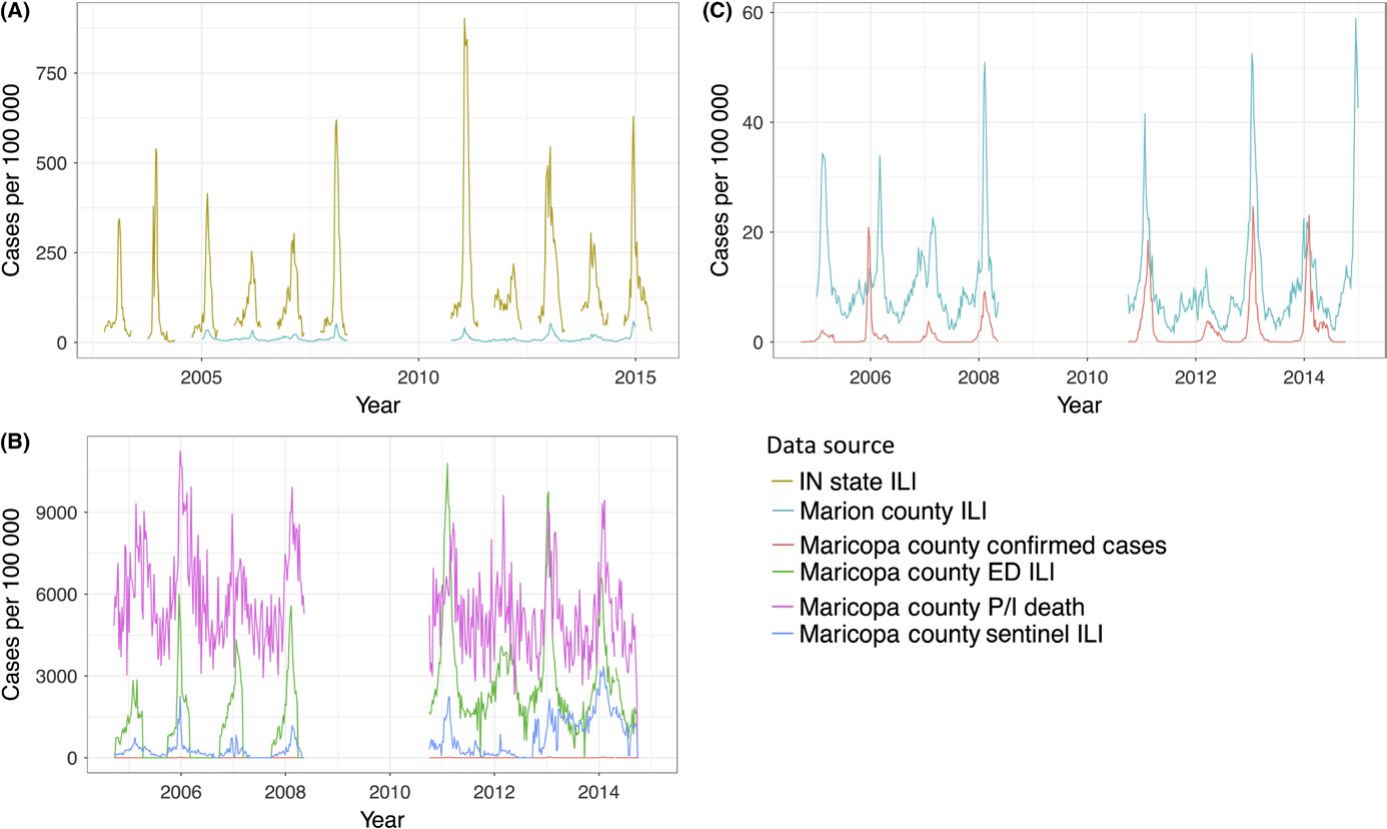
Influenza observations from 6 data streams Plot A, shows two data streams from Indiana: Indiana State and Marion County; plot B, shows 4 data streams from Maricopa County: confirmed cases, emergency department (ED) ILI, pneumonia and influenza (P/I) deaths, and sentinel ILI; and plot C, shows two data streams with the lowest case counts from A and B. 2008‐2010 pandemic flu seasons are excluded

Observations of each data stream are plotted in Figure 1. Timing of epidemic peaks lines up fairly well for all data types from a given region (Pearson's correlation coefficient for peak week by season ranging from 0.68 to 0.94 for all Maricopa County data types; Pearson's correlation coefficient 0.95 for the two Indiana data types); however, timing between locations varies. This is not unexpected given that influenza outbreaks do not peak simultaneously across the United States.^29^ Further, intensity of the peaks and extent of noise in the data varied substantially, both within and between locations. Maricopa County mortality data were particularly noisy, fluctuating widely throughout the year (lag‐one autocorrelation 0.560, compared to >0.85 for all other data types); although seasonal outbreak peaks are still visible, the signal is much weaker. In addition, background, or summer, levels differ greatly between data types and locations.

### 3.2 Optimal parameter values

Optimal parameter values for each data and error type are listed in Table 2. Forecast accuracy varied significantly based on the values chosen for scaling, OEV, and inflation factor, and different parameter combinations yielded optimal forecasts for each data type (Table 3). In particular, optimal scaling values varied substantially between data types, ranging from 0.2 to 0.3 for Maricopa County emergency department ILI to 250‐750 for Maricopa County laboratory‐confirmed cases. OEV and lambda were less sensitive to data type, as several different OEV and lambda values often performed similarly.

**Table 2:** Optimal parameter values for each data and error type

Data stream	Error type	OEV	Lambda	Scaling
Indiana State ILI	RMSE	0	1, 1.01	0.5
	MAPE	0	1, 1.01, 1.02	0.5
	Correlation	0	1, 1.01, 1.02	0.5
	Peak timing	0, 1, 2	1, 1.01, 1.02, 1.03	0.5
	Peak intensity	1, 2	1, 1.01	0.5
Marion County ILI	RMSE	0	1, 1.01	30
	MAPE	0	1, 1.01	30
	Correlation	0	1, 1.01	30
	Peak timing	0, 1, 2	1, 1.01, 1.02, 1.03	30
	Peak intensity	0	1, 1.01	30
Maricopa ED ILI	RMSE	2	1.01, 1.02, 1.03	0.3
	MAPE	2	1.01, 1.02, 1.03	0.3
	Correlation	2	1.01, 1.02, 1.03	0.2, 0.3
	Peak timing	2	1.01, 1.02, 1.03	0.2, 0.3
	Peak intensity	2	1.01, 1.02	0.2, 0.3
Maricopa sentinel ILI	RMSE	0	1	2, 3
	MAPE	0, 1	1, 1.01, 1.02, 1.03	2, 3
	Correlation	0, 1	1, 1.01, 1.02, 1.03	1, 2, 3
	Peak timing	1	1, 1.01, 1.02	1
	Peak intensity	1	1, 1.01, 1.02, 1.03	1
Maricopa lab	RMSE	0, 1	1	500, 750
	MAPE	0, 1	1	500, 750
	Correlation	2	1	250
	Peak timing	1, 2	1	250
	Peak intensity	2	1	750
Maricopa deaths	RMSE	1	1.01,	0.4
	MAPE	1	1.01, 1.02	0.4
	Correlation	1, 2	1.01, 1.02	0.3, 0.4
	Peak timing	1, 2	1.01, 1.02	0.3, 0.4
	Peak intensity	1	1.01	0.4

**Table 3:** Accuracy proportions by lead time using optimal parameters listed in Table 2, and comparative assessments based on historical expectance and likelihood

Data type	Accuracy proportion									
	Peak timing					Peak intensity				
	[−6, −4]	[−3, −1]	[0, 2]	Historical expectance	Historical likelihood	[−6, −4]	[−3, −1]	[0, 2]	Historical expectance	Historical likelihood
Indiana State ILI	16%‐25%	31%‐47%	51%‐69%	18.2%	18.2%	26%‐37%	21%‐27%	61%‐73%	18.2%	14.5%
Marion County ILI	26%‐36%	32%‐43%	70%‐97%	14.3%	14.3%	14%‐28%	45%‐73%	92%‐100%	14.3%	9.5%
Maricopa ED ILI	30%‐37%	31%‐39%	71%‐81%	12.5%	14.3%	23%‐32%	38%‐55%	86%‐93%	25%	14.3%
Maricopa sentinel ILI	16%‐27%	53%‐55%	55%‐71%	0%	32.1%	12%‐26%	35%‐68%	85%‐96%	0%	16.1%
Maricopa lab	38%‐41%	47%‐67%	82%‐87%	62.5%	35.7%	5%	30%	80%	0%	10.7%
Maricopa deaths	18%‐25%	29%‐38%	67%‐89%	0%	14.3%	71%	56%	86%	87.5%	73.2%

In addition to varying by data type, optimal parameter values were found to differ by accuracy metric assessed (RMSE, correlation, peak timing error, and peak intensity error). Of the 6 data types, only Maricopa County ED ILI, Maricopa County P&I deaths, and Marion County ILI yielded a single combination of parameter values that performed best according to all accuracy metrics (scaling 0.3, OEV 2, and lambda 1.01 for emergency department ILI; scaling 0.4, OEV 1, and lambda 1.01 for P&I deaths; scaling 30, OEV 0, and lambda 1.00 and 1.01 for Marion County ILI; Table 2). For all other data types, different parameter combinations performed best for different accuracy metrics.

### 3.3 Forecast accuracy by data type

**Figure 2:**
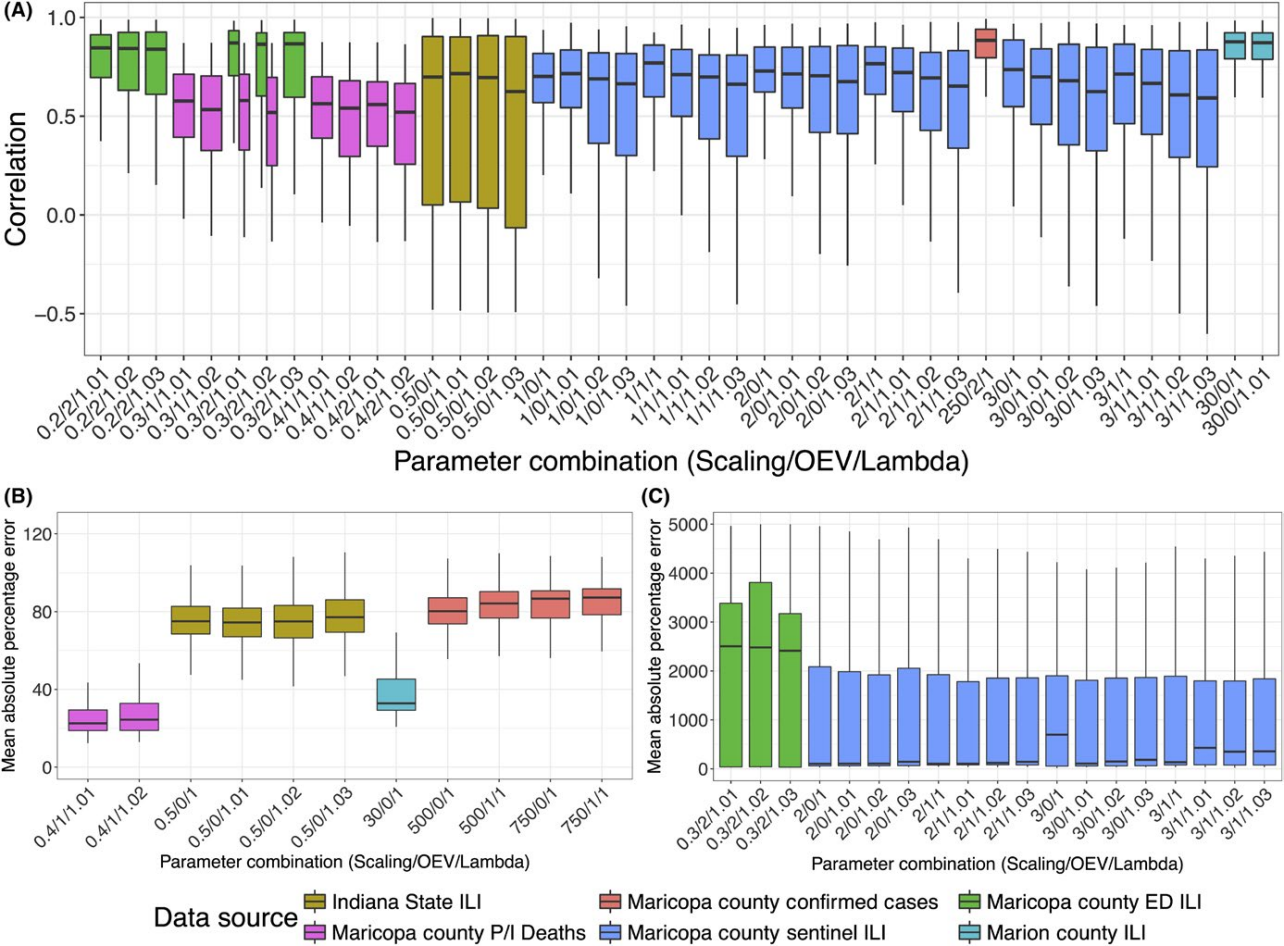
Boxplots of correlation and MAPE Plot A, shows correlations and pots B and C, show mean absolute percentage error (MAPE) between the posterior distribution and the observed data for each parameter combination used to run the forecast

Data type(s) yielding the most accurate forecasts depended on the accuracy metric examined. MAPE over the forecasting period was significantly higher for Maricopa ED ILI and sentinel ILI than all other data types (*P* < 5e‐8, Tukey's HSD; Figure 2a,b). Correlations between observed and forecasted values yielded similar results, with correlations being highest for Marion County data and for Maricopa County laboratory‐confirmed case data (*P* < 0.0005, Tukey's HSD; Figure 2c). Proportion of forecasts predicting peak timing within 1 week of the true value was also highest for Maricopa County laboratory‐confirmed cases (*P* < 7.5e‐7, chi‐square test of equal proportions; Table 3). Proportion of forecasts accurately predicting peak intensity, however, was highest for Maricopa County P&I deaths (*P* < 2e‐15, chi‐square test of equal proportions).

No significant association (by Spearman's rank correlation) was observed between lag‐one autocorrelation of a data stream (a measure of signal smoothness) and overall forecast accuracy for any of the 4 metrics used here. However, we note that, excluding Maricopa County P&I deaths, all data contained very little noise (lag‐one autocorrelation > 0.85), perhaps obscuring potential significant relationships between signal clarity and forecast accuracy.

## 4 DISCUSSION

In this study, we assess how data type and the associated use of 3 system inputs, namely, scaling, OEV, and an inflation factor against filter divergence, impact forecast accuracy. We compared results across a variety of data types (ILI on multiple spatial scales, mortality, and laboratory‐confirmed cases) over several influenza outbreaks. Overall, we found that forecast accuracy differed significantly both by data type and by choice of input values, particularly scaling; however, no clear, overarching patterns were observed between input value choices and accuracy. We conclude that making input choices in the absence of retrospective forecast and calibration is unintuitive. Specifically, without guidance gained through prior analysis on past seasonal data, scaling parameters in particular are less likely to be chosen appropriately.

Whereas both lambda and OEV were found to influence forecast accuracy, their impacts were relatively small and inconsistent. Scaling, meanwhile, was much more critical to forecast accuracy and varied by data type, ranging from 0.2 to 0.3 (for Maricopa ED ILI) to 100‐200 (for Maricopa laboratory‐confirmed cases). These results are consistent with those of Moss et al, who found that the range of observation probabilities (analogous to our scaling values) that yield optimal forecasts differs by data type.^26^ A range of factors influence appropriate choice of scaling, including overall number of people diagnosed and health‐seeking behavior for both influenza‐related and non‐influenza‐related illnesses. Past work suggests that forecasts are most accurate when data are scaled such that between 15% and 50% of a model population of size (100 000) are infected over the course of an epidemic. However, in a real‐time forecast, eventual attack rate of the current epidemic will not be known. In this case, appropriate scaling values must be chosen based on results from past retrospective forecasts using the same data stream, with input of experienced modelers, who possess requisite knowledge of the model, its assumptions, and its outputs. Uninformed scaling choices can potentially lead to public health actions that are not optimized at best and harmful at worst, negatively impacting both population health and public trust. This risk is particularly high in the case of novel data streams for which little previous knowledge of appropriate scaling values exists. A parallel case where experts are necessary for forecast quality control is described in Supporting information.

Previous studies have shown that in skillful forecasts, accuracy of forecast outcome increases as lead time decreases. Early in the influenza season, the model does not forecast outcomes well and contains high uncertainty on timing and intensity of the peak. However, as the model is trained with more influenza observations, forecast uncertainty decreases.^13^, ^15^ As expected, this pattern is observed here across spatial scales and data types (Table 3). However, overall forecast accuracy, as well as accuracy over time, was found to differ significantly by data type, although which data type performed best was inconsistent across different measures of forecast accuracy. Discussion on potential effects of noisy data on forecast accuracy is available in Supporting information.

We also explored the role of outcome metric of interest on ideal system input choice (ie, scaling/OEV/lambda), but found no consistent patterns. The current study suggests that system inputs are unlikely to systematically increase the accuracy of one forecast metric (eg, peak timing) at the expense of another. However, given that different individuals and organizations may place more value on certain outcome metrics over others, any role played by choice of scaling, lambda, or OEV should be identified so that modelers can preferentially enhance forecast accuracy of the desired metric.

We acknowledge there are several important limitations. First, we have data from only two locations. This may have hindered our ability to identify overarching patterns in forecast accuracy by data type and outcome metric, and to generalize results found here to other locations. However, we note that locations included in this study do not possess any obvious characteristics that would make the present results ungeneralizable. Future work should consider data from a much larger range of sources not only to increase the chances of identifying any consistent influence the user‐specified parameters have on forecast accuracy, but also to elucidate geographic resolutions at which forecasts can be generated and operationalized. A geographic resolution that is too fine may not exhibit an epidemic curve that can be modeled and forecasted. However, forecasts generated at larger spatial scales, while potentially critical for responses at these larger scales, may not be useful at the local level. Therefore, continued work testing the feasibility of parameter choice and generating forecasts using a variety of local data streams is needed.

We are also limited in that we cannot know the trajectory of the true influenza outbreaks in our states and counties of interest, and therefore must compare our forecasts to observed data. A forecast may thus be considered accurate while misrepresenting true influenza dynamics if the data streams themselves do not accurately reflect the “true” situation. Unfortunately, no gold standard estimate of the “true” epidemic exists, and combining different data to produce an aggregate estimate may actually lead to less accurate forecasts.^26^ However, in this study, all tested data streams were relatively smooth, and data streams from the same locations tended to peak at or around the same week, indicating that none of the data types was particularly noisy or obviously differentiated from the other data types. Furthermore, we emphasize that a forecast does not have to accurately represent the true influenza outbreak trajectory to be useful. A system accurately forecasting the number of hospitalized cases, for example, could be of great value to medical professionals, even if it underestimates the true, total number of influenza cases.

The present study finds that producing accurate forecasts is achievable using a variety of data types. Additionally, no consistent patterns in roles played by scaling, lambda, and OEV choice on forecast accuracy were observed by data type or outcome measure. Whereas the effects of lambda and OEV were found to be small, an appropriate scaling value is critical to forecast accuracy. We therefore recommend that forecasts using novel data streams be generated with a range of realistic scaling values informed by retrospective forecasts of past influenza seasons. Additionally, experienced modelers should be heavily involved not only with the choice of an appropriate range of scaling values, but also with overall forecast development and analysis.

## CONFLICT OF INTEREST

J.S. discloses partial ownership of SK Analytics. All other authors declare no competing interests.
