# Supplementary material for: Influenza forecast optimization when using different surveillance data types and geographic scale
Source: Influenza Other Respir Viruses. 2018 Aug 21;12(6):755–64. doi: 10.1111/irv.12594 (PMC6185890; doi:10.1111/irv.12594)
Supplement: Supplementary file 1 [file IRV-12-755-s001.docx]

SUPPLEMENTAL MATERIALS

**Supplementary Figure 1.**


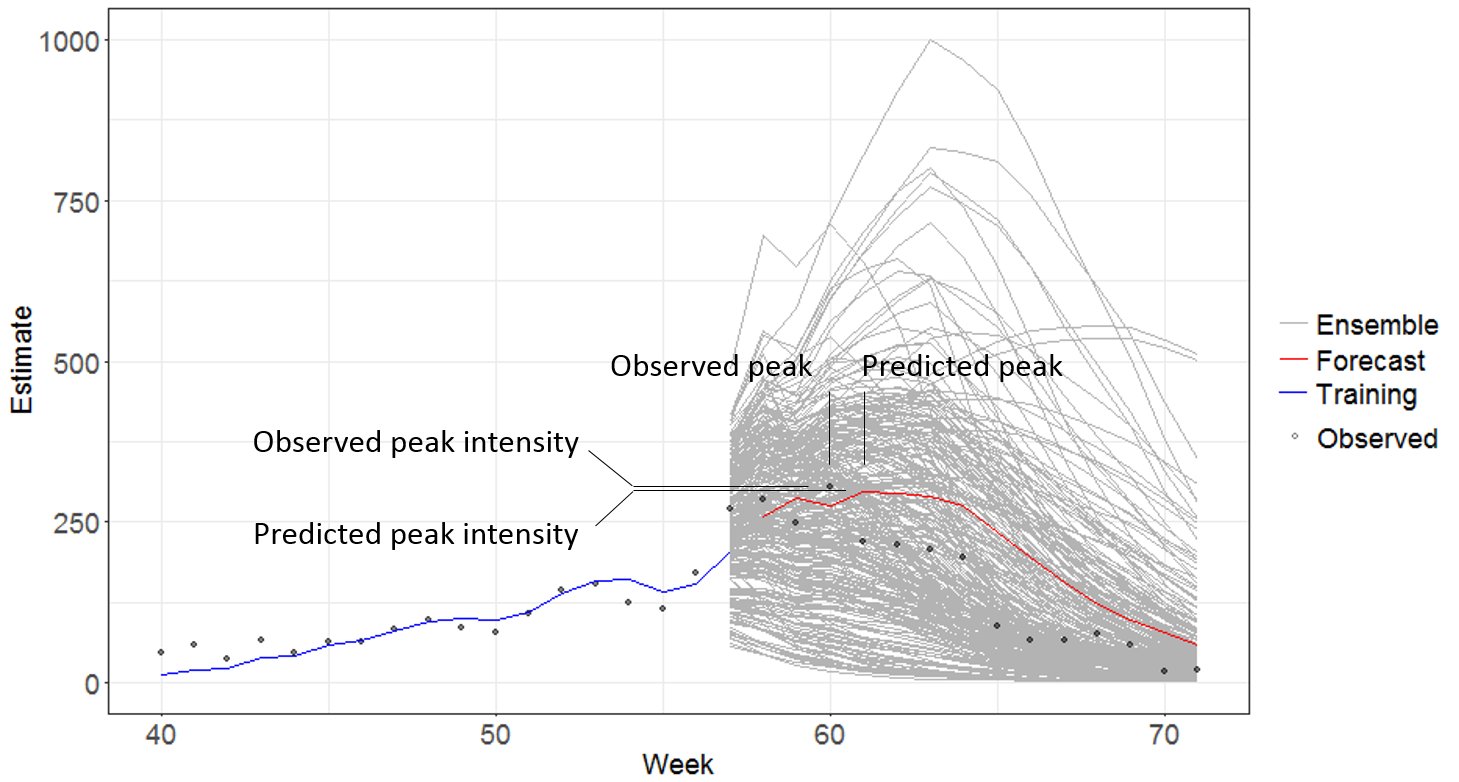


This figure shows a single 300-member run for Indiana State for the 2006-2007 flu season, using scaling of 0.5, Lambda of 1.01, and OEV of 10. The blue line shows the weeks where the model was trained to the observed flu incidence. In this case, the model is trained through week 56 and the forecast (mean trajectory in red) begins at week 57. The red line is the mean of the individual ensemble members (in gray), which represent possible outcomes of the flu outbreak. The forecast was run as if we did not have any observations past week 56.

**Additional Methods**

*ILI Data Processing*

While influenza cases predominantly occur during wintertime in the US, diagnoses of ILI associated with other respiratory infections, occur throughout the year, including during summer months. This summertime, baseline level of ILI is assumed to persist during the winter influenza season. To remove this baseline, we subtracted the 60^th^ percentile of ILI observations from June to August (MMWR weeks 22-34) from all ILI observations. This subtraction eliminates background noise and provides a cleaner signal of influenza-specific ILI cases.[^1^](#_ENREF_1) ILI data from the Indiana State Department of Health were available only from MMWR weeks 40 through 20 (no summertime values), so we could not calculate an offset value. For these time series, we used the raw data values.

*Processing for All Data*

Each data stream was converted to rate data before being incorporated into the model. Weekly Maricopa County and Indiana State ILI data were divided by the total number of patient visits each week and then scaled to represent ILI cases per 100,000 patient visits. Weekly P&I deaths were similarly normalized using weekly total deaths reported to represent P&I deaths per 100,000 total deaths. Total patient visits were not provided for Marion County, so Marion County ILI was normalized using the average county population in 2000 and 2016 (9 million) to represent ILI cases per 100,000 people.[^2^](#_ENREF_2)^,^[^3^](#_ENREF_3) Maricopa County laboratory-confirmed influenza cases were normalized using the average county population of 4 million to represent laboratory confirmed cases per 100,000 people.[^4^](#_ENREF_4)

*SIRS Model*

The equations for the model are:

$\frac{dS}{dt}= \frac{N-S-I}{L}- \frac{\beta\left( t \right)IS}{N}- \alpha$ (1)

$\frac{dI}{dt}= \frac{\beta\left( t \right)IS}{N}-\frac{I}{D}+ \alpha$ (2)

where *S* is the number of susceptible people in the population, *t* is time in years, *N* is the population size, *I* is the number of infectious individuals, *N-S-I* is the number of recovered individuals, *β(t)* is the contact rate at time *t*, *L* is the average duration of immunity, and *D* is the mean infectious period.

Contact rate, *β(t)*, is defined by $\beta\left( t \right)= {R_{0}(t)}/D$, in which the basic reproductive number, $R_{0}(t)$, is the number of secondary infections that originate from the average infectious person in a fully susceptible population at time *t*.

Transmission rates are modulated by absolute humidity (AH), which adjusts$R_{0}(t)$ through an exponential relationship similar to how AH has been shown to affect both influenza virus survival and transmission in laboratory experiments:[^5^](#_ENREF_5)

$R_{0}\left( t \right)= R_{0 min}+\left( R_{0 max}-R_{0 min} \right)e^{-aq(t)}$ (3)

In this equation, $R_{0 min}$ is the minimum daily basic reproductive number, $R_{0 max}$ is the maximum daily basic reproductive number, *a* = 180, and *q(t)* is daily specific humidity, a measure of AH. The value of *a* is estimated from the laboratory regression of influenza virus survival upon AH.[^5^](#_ENREF_5) The model was run deterministically such that transitions between model states were calculated directly from Equations 1 and 2, and with an influenza importation rate, α, of 0.1 infections per day (1 infection every 10 days).

*Model-Assimilation Methods*

During the model training period, the filter adjusts the model state space and parameters to better reflect observed incidence and capture characteristics of the local outbreak. Upon reaching each successive observation, integration is halted and EAKF uses the latest observation to update the observed system state. Cross ensemble covariability is then used to deterministically adjust unobserved state variables and parameters (see reference 28 in main References for details on the algorithm).

Kalman filters require estimates of the mean and covariance matrix, which can be computed directly from the distribution of ensemble states. In addition, ensemble filters store information about observable and non-observable parameters and their inter-variable relationships in model solutions (i.e. individual ensemble members), which are potential realizations of outbreak dynamics based on initial parameters and observations. Further details on EAKF can be found in Shaman and Karspeck.[^1^](#_ENREF_1)

*Choosing Optimal Parameter Sets*

In addition to evaluating peak timing and intensity accuracy, root mean square error (RMSE), mean absolute percentage error (MAPE), and a simple correlation were calculated for each forecast generated with each parameter set. Specifically, for each forecast, the forecasted values (the red line in Figure S1) are compared to the observed values (black points in Figure S1) for the duration of the forecast period. In other words, we compare a time series of forecasted values to a time series of observed values. RMSE is calculated as:

$$RMSE=\sqrt{\frac{\sum_{t=1}^{T} {(F_{t}-O_{t})}^{2}}{T}}$$

where *F_t_* is the forecasted value at time *t*, *O_t_* is the observed value at time *t*, and *T* is the total number of weeks for which forecasted values are generated (which will be greater the earlier in the season the forecast is started). Unlike RMSE, MAPE controls for the difference in magnitude of the observations from different data streams, and is thus used when comparing various data types to each other. MAPE is calculated as:

$$MAPE=\frac{100\%}{T}\sum_{t=1}^{T} \left| \frac{O_{t}-F_{t}}{O_{t}} \right|$$

Thus, for each forecast using a given parameter set, RMSE, MAPE, and correlation each yield a single value describing how closely the forecasted values correspond to the observed values.

*Historical Expectancy/Likelihood*

In order to determine whether our forecasts offered value above and beyond what could be inferred from the historical data alone, we calculated peak timing and peak intensity accuracy using two purely historical measures. More specifically, historical expectance was calculated for a given season by first removing that season from the dataset. Then the predicted peak timing was set as the mode of the peak timing values for all other season in the dataset, and the predicted peak intensity was set as the median of the remaining seasons’ peak intensity values. If no single mode peak timing existed, the median was used instead. As in the main text, forecasts were considered to be accurate if the predicted peak timing was within one week of the observed value, and if the predicted peak intensity was within 25% of the observed value. The second approach, historical likelihood, considered the full distribution of peak timing and intensity values after removing a given season from the dataset. Forecast accuracy for a given season was then calculated as the proportion of values in the peak timing and intensity distributions that yielded accurate forecasts. Both historical expectance and likelihood were calculated separately for each data type.

As seen in Table 3, our forecasts generated using optimal parameters and the SIRS-EAKF framework, almost always outperform both historical forecast methods several weeks before the outbreak peak, further supporting the usefulness of these and similar forecasts.

*Cross-Validation of Optimal Parameter Sets*

To determine whether optimal parameter sets were consistent across seasons, we performed leave-one-out cross validation, wherein optimal parameters by each of our four metrics of interest (peak timing accuracy, peak intensity accuracy, RMSE, and correlation) were determined for each data type after removing each individual season in turn. In general, optimal parameter sets remained fairly consistent regardless of which season was removed, and in the majority of cases, common optimal parameter sets could be found across all seasons (Supplementary Table 1). Thus, we have reason to believe that parameter sets obtained using the methods outlined in the main text will also be optimal, or close to optimal, for forecasts generated with seasonal data obtained for future outbreaks.

**Additional Discussion**

*Noisy data*

Given previous findings that more regular epidemic data (i.e., data lacking substantial noise) yield more accurate forecasts,[^6^](#_ENREF_6) it is interesting that we do not observe this pattern here. No significant correlation was found between data lag-one autocorrelation and forecast accuracy for any of the four metrics assessed. Furthermore, Maricopa county P&I death data, by far the noisiest data stream used here (lag-one autocorrelation of 0.560), yielded the most accurate forecasts of peak intensity, and was among the best-performing data types for RMSE. In addition, the strength and regularity of the epidemic signal did not contribute to parsimony in parameter choice: a single optimal set of parameter values could only be identified for Maricopa county ED ILI and P&I death data. Thus, when many local data streams are available for forecasting, it may not be intuitive which will yield the most accurate forecasts.

*Quality control parallel*

The necessity of involving experts as a measure of forecast quality control is not unique to influenza forecasting. A comparison may be drawn here to numerical weather predictions, which use meteorological observations, models, and data assimilation methods to produce an ensemble of predictions for both short- and long-term forecasts.[^7^](#_ENREF_7)^,^[^8^](#_ENREF_8) Weather predictions are generated at operational centers such as the National Centers for Environmental Prediction of the National Weather Service, by individuals who are trained in the various models and methods used to generate predictions, maintain quality control, assess forecast skillfulness over many years, and improve upon current systems. Reasonably, operational influenza forecasts should be run and maintained in a similar manner by those who have expertise in prediction and quality control methods.

| **Supplementary Table 1.**  Optimal parameter sets obtained for each data type and accuracy metric when the season indicated is removed from the forecast results. If a single common parameter set is ideal regardless of the season removed, it is indicated in the “consensus” column. | | | | | | | | | | | | |
| --- | --- | --- | --- | --- | --- | --- | --- | --- | --- | --- | --- | --- |
|  | **02-03** | **03-04** | **04-05** | **05-06** | **06-07** | **07-08** | **10-11** | **11-12** | **12-13** | **13-14** | **14-15** | **Consensus** |
| **Indiana State ILI** | | | | | | | | | | | | |
| Peak Timing | 0.5  0/1/2  00/01/02 | 0.5  2  00 | 0.5  0  01 | 0.5  0/2  00/01/02 | 0.5  1/2  00 | 0.5  0/1/2  00/01/02 | 0.5  2  00 | 0.5  0  01/02 | 0.5  0/1/2  00/01/02/03 | 0.5  2  00 | 0.5  2  00 | N/A |
| Peak Intensity | 0.5  2  00 | 0.5  1  01 | 0.5  2  00 | 0.5  1  01 | 0.5  2  00 | 0.5  2  00 | 0.5  2  00 | 0.5  1  01 | 0.5  1  01 | 0.5  1  01 | 0.5  2  00 | N/A |
| RMSE | 0.5  0  01 | 0.5  0  01 | 0.5  0  00/01 | 0.5  0  00/01 | 0.5  0  00/01 | 0.5  0  01 | 0.5  0  01 | 0.5  0  01 | 0.5  0  01 | 0.5  0  01 | 0.5  0  01 | 0.5  0  01 |
| MAPE | 0.5  0  00/01/02 | 0.5  0  00/01 | 0.5  0  00/01/02 | 0.5  0  00/01/02 | 0.5  0  00/01/02 | 0.5  0  00/01 | 0.5  0  00/01/02 | 0.5  0  00/01/02 | 0.5  0  00/01 | 0.5  0  00/01/02 | 0.5  0  00/01 | 0.5  0  00/01 |
| Correlation | 0.5  0  01 | 0.5  0  01 | 0.5  0  01/02 | 0.5  0  01/02 | 0.5  0  01 | 0.5  0  01 | 0.5  0  00/01/02 | 0.5  0  00/01/02 | 0.5  0  01 | 0.5  0  01 | 0.5  2  00 | N/A |
| **Marion County ILI** | | | | | | | | | | | | |
| Peak Timing | NA | NA | NA | 30  1/2  00/01/02 | 30  0/1/2  00/01/02/03 | 30  0/1/2  00/01/02/03 | 30  0/1/2  00/01/02/03 | 30  0/1/2  00/01/02/03 | 30  0/1/2  00/01/02 | 30  1  00/01/02 | NA | 30  1  00/01/02 |
| Peak Intensity | NA | NA | NA | 30  0  00/01 | 30  0  00/01 | 30  0  00/01 | 30  0  00/01 | 30  0  00/01 | 30  0  01 | 30  0  01 | NA | 30  0  01 |
| RMSE | NA | NA | NA | 30  0  00/01 | 30  0  00/01 | 30  0  00/01 | 30  0  00/01 | 30  0  00/01 | 30  0  00/01 | 30  0  00/01 | NA | 30  0  00/01 |
| MAPE | NA | NA | NA | 30  0  00/01 | 30  0  00/01 | 30  0  00/01 | 30  0  00/01 | 30  0  00/01 | 30  0  00/01 | 30  0  00/01 | NA | 30  0  00/01 |
| Correlation | NA | NA | NA | 30  0  00/01 | 30  0  00/01 | 30  0  00/01 | 30  0  00/01 | 30  0/1  00/01 | 30  0  00/01 | 30  0  00/01 | NA | 30  0  00/01 |
| **Maricopa ED ILI** | | | | | | | | | | | | |
| Peak Timing | NA | NA | 0.2/0.3  2  01/02/03 | 0.2/0.3  2  01/02/03 | 0.2/0.3  2  01/02/03 | 0.2/0.3  2  01/02/03 | 0.2/0.3  2  01/02/03 | 0.2/0.3  2  01/02/03 | 0.2/0.3  2  01/02/03 | 0.2/0.3  2  01/02/03 | NA | 0.2/0.3  2  01/02/03 |
| Peak Intensity | NA | NA | 0.2/0.3  2  01/02/03 | 0.2/0.3  2  01/02/03 | 0.3  2  01/02 | 0.3  2  01/02 | 0.2/0.3  2  01/02/03 | 0.2/0.3  2  01/02 | 0.2/0.3  2  01/02 | 0.2/0.3  2  01/02/03 | NA | 0.3  2  01/02 |
| RMSE | NA | NA | 0.3  2  01/02/03 | 0.3  2  01/02/03 | 0.3  2  01/02/03 | 0.3  2  01/02/03 | 0.3  2  01/02/03 | 0.3  2  01/02/03 | 0.3  2  01/02/03 | 0.3  2  01/02/03 | NA | 0.3  2  01/02/03 |
| MAPE | NA | NA | 0.3  2  01/02/03 | 0.3  2  01/02/03 | 0.3  2  01/02/03 | 0.3  2  01/02/03 | 0.3  2  01/02/03 | 0.3  2  01/02/03 | 0.3  2  01/02/03 | 0.3  2  01/02/03 | NA | 0.3  2  01/02/03 |
| Correlation | NA | NA | 0.2/0.3  2  01/02/03 | 0.3  2  01 | 0.2/0.3  2  01/02/03 | 0.2  2  01 | 0.3  2  01 | 0.2/0.3  2  01/02/03 | 0.3  2  01 | 0.3  2  01 | NA | NA |
| **Maricopa Sentinel ILI** | | | | | | | | | | | | |
| Peak Timing | NA | NA | 1  0/1  00/01/02 | 1  1  00/01 | 1/3  0/1  00 | 1  1  00 | 3  0  00/01/02/03 | 1/2/3  1  00/01/02 | 1/3  0/1  00 | 2/3  0/1  00 | NA | NA |
| Peak Intensity | NA | NA | 1/2/3  1  00/01/02/03 | 1/2  1  00/01/02/03 | 1/2  1  00/01/02/03 | 1/2/3  1  00/01/02/03 | 2/3  0/1  00/01/02/03 | 1/2  1  00/01/02/03 | 1/2  1  00/01/02/03 | 2  0/1  00 | NA | 2  1  00 |
| RMSE | NA | NA | 3  0  00 | 3  0  00 | 3  0  00 | 3  0  00 | 3  0  00 | 3  0/1  00 | 3  0  00 | 3  0  00 | NA | 3  0  00 |
| MAPE | NA | NA | 2/3  0/1  00/01/02/03 | 2/3  0/1  00/01/02/03 | 2/3  0/1  00/01/02/03 | 2/3  0/1  00 | 2/3  0/1  00/01/02/03 | 2/3  0/1  00/01/02/03 | 2/3  0/1  00/01/02/03 | 2/3  0/1  00/01/02/03 | NA | 2/3  0/1  00 |
| Correlation | NA | NA | 1/2/3  0/1  00 | 1/2/3  0/1  00 | 1/2/3  0/1  00 | 1/2/3  0/1  00 | 1/2/3  0/1  00 | 1/2/3  0/1  00 | 1/2/3  0  00/01 | 1/2/3  0/1  00 | NA | 1/2/3  0  00 |

| **Maricopa Lab** | | | | | | | | | | | | |
| --- | --- | --- | --- | --- | --- | --- | --- | --- | --- | --- | --- | --- |
| Peak Timing | NA | NA | 250  1/2  00 | 250  1/2  00 | 250  1/2  00 | 250  1/2  00 | 250/750  1/2  00 | 250  0/1/2  00 | 250/750  2  00 | 250/750  0/1/2  00 | NA | 250  2  00 |
| Peak Intensity | NA | NA | 750  2  00 | 750  1/2  00 | 250/500/750  2  00 | 750  2  00 | 750  2  00 | 250/500/750  2  00 | 750  1/2  00 | 750  1/2  00 | NA | 750  2  00 |
| RMSE | NA | NA | 500/750  0/1  00/01/02/03 | 500/750  0/1  00/01/02/03 | 500/750  0/1  00/01/02/03 | 500/750  0/1  00/01/02/03 | 500/750  0/1  00 | 500/750  0/1  00/01/02/03 | 500/750  0/1  00/01/02/03 | 500/750  0/1  00 | NA | 500/750  0/1  00 |
| MAPE | NA | NA | 500/750  0/1  00/01 | 500/750  0/1  00 | 500/750  0/1  00 | 500/750  0/1  00/01 | 500/750  0/1  00 | 500/750  0/1  00 | 500/750  0/1  00 | 500/750  0/1  00 | NA | 500/750  0/1  00 |
| Correlation | NA | NA | 250/500  1  00/01 | 250/500  1/2  00 | 250/500  1  00 | 250/500  1  00 | 250/500  1  00 | 250/500  1  00 | 250/500  0/1/2  00 | 250/500  1  00 | NA | 250/500  1  00 |
| **Maricopa Deaths** | | | | | | | | | | | | |
| Peak Timing | NA | NA | 0.3/0.4  1/2  01/02 | 0.3/0.4  1/2  01/02 | 0.3/0.4  1/2  01/02 | 0.3/0.4  1/2  01/02 | 0.3/0.4  1/2  01/02 | 0.3/0.4  1/2  01/02 | 0.3/0.4  1/2  01/02 | 0.3/0.4  1/2  01/02 | NA | 0.3/0.4  1/2  01/02 |
| Peak Intensity | NA | NA | 0.4  1  01 | 0.4  1  01 | 0.4  1  01 | 0.4  1  01 | 0.4  1  01 | 0.4  1  01 | 0.4  1  01 | 0.4  1  01 | NA | 0.4  1  01 |
| RMSE | NA | NA | 0.4  1  01/02 | 0.4  1  01/02 | 0.4  1  01/02 | 0.4  1  01/02 | 0.4  1  01/02 | 0.4  1  01/02 | 0.4  1  01/02 | 0.4  1  01/02 | NA | 0.4  1  01/02 |
| MAPE | NA | NA | 0.4  1  01 | 0.4  1  01 | 0.4  1  01 | 0.4  1  01 | 0.4  1  01 | 0.4  1  01 | 0.4  1  01 | 0.4  1  01 | NA | 0.4  1  01 |
| Correlation | NA | NA | 0.3/0.4  1/2  01 | 0.3/0.4  1/2  01 | 0.3/0.4  1/2  01 | 0.3/0.4  1  01 | 0.3/0.4  1/2  01 | 0.3/0.4  1/2  01 | 0.3/0.4  1/2  01 | 0.3/0.4  1/2  01 | NA | 0.3/0.4  1  01 |

Rows within each cell corresponds to values for scaling, OEV, and then lambda. Decimal values only are shown here for lambda.

**References**

1. Shaman J, Karspeck A. Forecasting seasonal outbreaks of influenza. *Proc Natl Acad Sci U S A.* 2012;109:20425-20430.

2. United States Census Bureau. Counties Ranked by Population: 2000. 2001. https://[www.census.gov/population/www/cen2000/briefs/phc-t4/tables/tab02.pdf](http://www.census.gov/population/www/cen2000/briefs/phc-t4/tables/tab02.pdf). Accessed 2016.

3. United States Census Bureau. QuickFacts Marion County, Indiana. 2016. https://[www.census.gov/quickfacts/fact/table/marioncountyindiana/POP010210](http://www.census.gov/quickfacts/fact/table/marioncountyindiana/POP010210). Accessed 2016.

4. United States Census Bureau. QuickFacts Phoenix City, Arizona. 2016. https://[www.census.gov/quickfacts/fact/table/phoenixcityarizona/PST045216](http://www.census.gov/quickfacts/fact/table/phoenixcityarizona/PST045216). Accessed 2016.

5. Shaman J, Kohn M. Absolute humidity modulates influenza survival, transmission, and seasonality. *Proc Natl Acad Sci U S A.* 2009;106:3243-3248.

6. Reis J, Shaman J. Retrospective Parameter Estimation and Forecast of Respiratory Syncytial Virus in the United States. *PLoS Comput Biol.* 2016;12:e1005133.

7. Barker D, Huang X-Y, Liu Z, et al. The weather research and forecasting model's community variational/ensemble data assimilation system: WRFDA. *Bull Am Meteorol Soc.* 2012;93:831-843.

8. Lynch P. The origins of computer weather prediction and climate modeling. *J Comput Phys.* 2008;227:3431-3444.
